# Phenolic Compounds in Bacterial Inactivation: A Perspective from Brazil

**DOI:** 10.3390/antibiotics12040645

**Published:** 2023-03-24

**Authors:** Angélica Correa Kauffmann, Vinicius Silva Castro

**Affiliations:** 1 Chemistry Department, Federal University of Mato Grosso, Cuiaba 78060-900, MT, Brazil; angelica.kauffmann@ufmt.br; 2 Department of Biological Sciences, University of Lethbridge, Lethbridge, AB T1K 3M4, Canada

**Keywords:** heterocyclic compounds, phenolic compounds, pyran, food microbiology, microbial pathogen

## Abstract

Phenolic compounds are natural substances that are produced through the secondary metabolism of plants, fungi, and bacteria, in addition to being produced by chemical synthesis. These compounds have anti-inflammatory, antioxidant, and antimicrobial properties, among others. In this way, Brazil represents one of the most promising countries regarding phenolic compounds since it has a heterogeneous flora, with the presence of six distinct biomes (Cerrado, Amazon, Atlantic Forest, Caatinga, Pantanal, and Pampa). Recently, several studies have pointed to an era of antimicrobial resistance due to the unrestricted and large-scale use of antibiotics, which led to the emergence of some survival mechanisms of bacteria to these compounds. Therefore, the use of natural substances with antimicrobial action can help combat these resistant pathogens and represent a natural alternative that may be useful in animal nutrition for direct application in food and can be used in human nutrition to promote health. Therefore, this study aimed to (i) evaluate the phenolic compounds with antimicrobial properties isolated from plants present in Brazil, (ii) discuss the compounds across different classes (flavonoids, xanthones, coumarins, phenolic acids, and others), and (iii) address the structure–activity relationship of phenolic compounds that lead to antimicrobial action.

## 1. Introduction

Few organisms have had such an influence on human history as microorganisms. This influence can be seen in the production of food such as beers, bread, cheeses, and fermented products; in drug production using bacteria as clonal vectors; and in the onset of diseases linked to these microorganisms. Although several microorganisms are a great ally to human beings in obtaining these foods and drugs, it is unquestionable that pathogenic microorganisms represent a strong opponent, with an impact on the lives of thousands of people over the centuries. As an example, we can mention that the adoption of antiseptic hygiene principles proposed by the then-physician Joseph Lister in 1867 [1] made him known as the father of modern surgery [2] due to the impact of the reduction in death rates after the adoption of these principles. After that, the discovery of penicillin by Alexander Fleming in 1928 brought a very powerful weapon to human beings in the fight against these pathogens [3]. During the following years, the investigation of new antibacterial compounds was exhaustively studied, and several new molecules were obtained for different classes of pathogens. However, as in any dispute, bacteria have developed different mechanisms to attenuate the power of these drugs, and we are currently living in the era of multiresistant microorganisms [4]. In this sense, the academic world works in different directions to solve these resistance mechanisms (or just to stop the spread of resistance genes), and in this way, the use of natural compounds can play a prominent role in the fight against these resistant microorganisms.

Brazil is an important country considering natural compounds since it has six biomes (Cerrado, Amazon, Atlantic Forest, Caatinga, Pantanal, and Pampa) [5], in addition to a vast number of plants, fruits, and native vegetables. Although there is still an immense number of substances to be discovered, several studies conducted recently in the country have elucidated compounds with antimicrobial potential, which could be used as one of the strategies to combat pathogenic bacteria. Among these compounds, we highlight the presence of phenolic groups. Phenolic compounds encompass many substances that are produced through the secondary metabolism of plants, fungi, and bacteria, in addition to being produced by chemical synthesis [6,7]. Studies indicate that there are several pharmacological activities associated with these compounds, including antibacterial activity [8,9]. Additionally, one of the main factors that suggest antibacterial action is related to the substituent groups contained in the phenolic compounds, which influences the increase in the lipophilicity of the molecule in the antibiofilm property and in the modulating action of antibiotics [10,11,12,13,14,15]. Therefore, our study addressed the different classes involving phenolic compounds from substances found in studies performed in Brazil.

## 2. Search Strategy

In this study, a literature search was performed using online databases: Google Scholar, Scifinder, PubMed, Wiley Online, Science Direct, and Federated Academic Community (CAFe—Brazil). The following terms were used: “Flavonoids AND Antibacterial activity AND Brazil”; “Coumarin AND Antibacterial Activity AND Brazil”; Phenolic Acids AND Antibacterial Activity AND Brazil”; “Lignans AND Antibacterial Activity AND Brazil”; “Anthraquinones AND Antibacterial activity AND Brazil”; “Xanthones AND Antibacterial Activity AND Brazil”; “Acetophenone AND Antibacterial Activity AND Brazil”; “Benzophenone AND Antibacterial Activity AND Brazil”; “Tannins AND Antibacterial Activity AND Brazil”; and “Phenylpropanoids AND Antibacterial Activity AND Brazil”. Only articles containing phenolic compounds isolated from plants analyzed in the Brazilian territory were considered in this study. Information about the name of the substance, the plant from which the phenolic compound originated, the microorganisms tested for antimicrobial action, the inhibitory concentration, and the location where the plant was collected (municipality and state of Brazil) are described in Table 1.

## 3. Phenolic Compounds

Phenolic compounds are substances widely produced by plants, with more than two hundred thousand compounds currently known. In their basic structure, phenolic compounds have a ring with all sp2-hybridized carbons, a planar structure with angles of 120° and electrons π delocalized, with one or more hydroxyl group bonds (O–H) [6,31,32,33]. This class of substances is divided into phenolic acids and polyphenols and can be found combined with other phenolic groups, mono- or polysaccharide structures, or occur as derivatives of esters and methyl esters [8,34].

Phenolic compounds are also aromatic molecules and play important roles in plant growth and reproduction, acting as allopathic agents. When induced by biotic and abiotic stress, they synthesize phytoalexin substances as a plant defense mechanism. Many phenolic compounds are attractive to pollinators in addition to being responsible for the organoleptic characteristics of vegetable foods [32,34,35,36].

**Figure 1:**
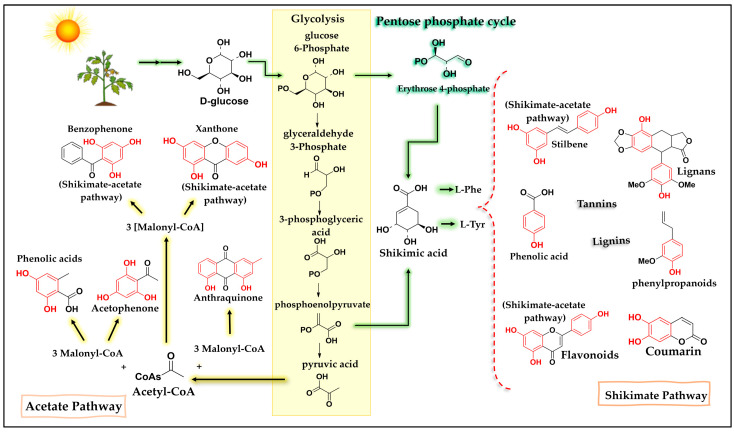
Simplified diagram of the shikimate and acetate pathways, which are responsible for the formation of phenolic compounds. Caption: The shikimate pathway occurs through the reaction between phosphoenolpyruvate and erythrose 4-phosphate, forming shikimic acid and leading to the formation of a variety of phenolic compounds. The acetate pathway occurs through the decarboxylation of pyruvic acid to form acetyl-CoA, which combines with malonyl-CoA units to form phenolic compounds.

In general, there are two metabolic pathways by which phenolic compounds can be synthesized, the *shikimate pathway* (1), which occurs through the combination of phosphoenolpyruvate (an intermediate of the glycolytic pathway) and erythrose 4-phosphate (from the pentose phosphate pathway), which generates shikimic acid, responsible for the formation of the amino acids, phenylalanine and tyrosine. After the formation of these amino acids, they undergo deamination and generate cinnamic acid and, thus, enter the phenylpropanoid pathway, producing compounds such as flavonoids, isoflavonoids, coumarins, lignans, lignins, and stilbenes. Another known pathway is the *acetate pathway* (2), responsible for the formation of several substances, including aromatic polyketides (anthraquinones, xanthones, benzophenones, and acetophenones) (Figure 1). The formation of phenolic compounds by this route occurs through the reaction of an acetyl-CoA unit, originating from glycolysis, and malonyl-CoA units following aldol reactions, Claisen condensation reactions, and enolization [37,38].

Thus, phenolic compounds are found in abundance in vegetables, fruits, and cereals [39]. The high consumption of foods rich in phenolic compounds can prevent a series of diseases [40]. Furthermore, studies indicate great biological potential associated with these compounds [7], including antiviral, antioxidant, antitumor, antiallergic, anti-inflammatory, fungal, and antibacterial activities [41,42,43,44,45,46,47]. In the next topics, we will address the compounds formed from these pathways.

## 4. Flavonoids

**Figure 2:**
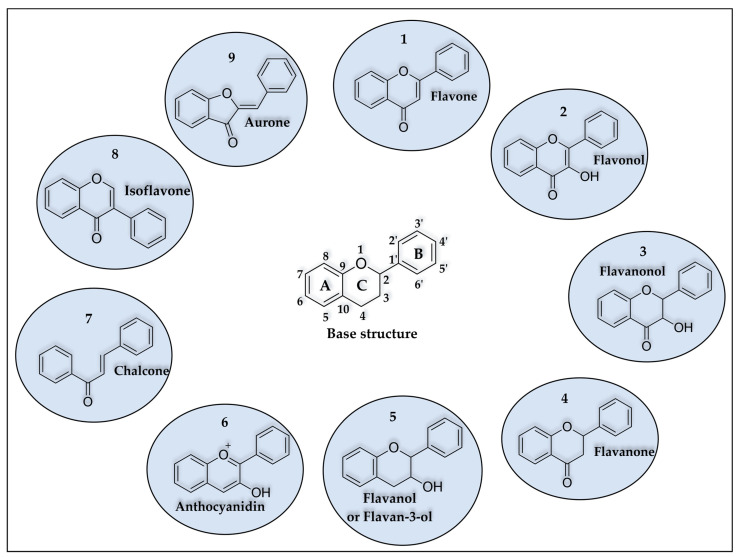
Base structure and classes of flavonoids. (**1**) flavone, (**2**) flavonol, (**3**) flavanonol, (**4**) flavanone, (**5**) flavanol, (**6**) anthocyanidin, (**7**) chalcone, (**8**) isoflavone, and (**9**) aurone.

Flavonoids are polyphenolic compounds biosynthesized from the shikimate and acetate pathways, and they are the class of phenolic compounds with the highest number of reported substances, with more than 4000 types of naturally occurring flavonoids [37,48]. Structurally, flavonoids contain two aromatic rings (A and B), with fifteen carbon atoms in their basic skeleton, connected by a bridge of three carbon atoms, arranged in C6-C3-C6, providing a third ring [49]. They are structures that differ in the saturation of the C ring, in the position of aromatic ring B on carbons C2 or C3, and in hydroxylation patterns [50], causing subclasses of flavonoids: flavonols, flavones, flavanones, flavanol, flavanonols, isoflavones, aurones, anthocyanins, and chalcones (Figure 2). The pattern of substitution can occur in flavonoids of natural origin, such as hydroxyl, methyl, phenyl, glycosides, aliphatic, isoprenyl, aromatic acids, and methoxyl [51,52].

In plants, these compounds are mainly responsible for the red, blue, and purple pigments that color them [50], have UV protection functions, modulate enzymatic activity, attract or repel insects, are attractive to pollinators, or act as antiviral, fungal, and bacterial protectors [53,54]. Several natural flavonoids show good bacterial inhibition, acting as bactericides and bacteriostatic agents; however, the biological activity depends on the substituent groups of the flavonoid structure, which vary between structures [55,56]. The inhibitory potential of flavonoids may vary according to the molecule analyzed and, although the factors that lead flavonoids to bacterial inhibition are not fully elucidated in the literature, several studies point to the participation of flavonoids in the disruption of cell membranes [57]. This disruption would be derived from the partitioning of nonpolar compounds in the hydrophobic interior of the membrane and due to the interaction between the bacterial membrane and the formation of hydrogen bonds between polar groups of cell lipids with the more hydrophilic flavonoids [57]. In addition, other studies have pointed to an increase in cell permeability through a decrease in bilayer lipids [58]. Additionally, other studies have demonstrated the ability to generate reactive oxygen species (ROS) that can cause alterations in membrane permeability and lead to membrane damage [59].

**Figure 3:**
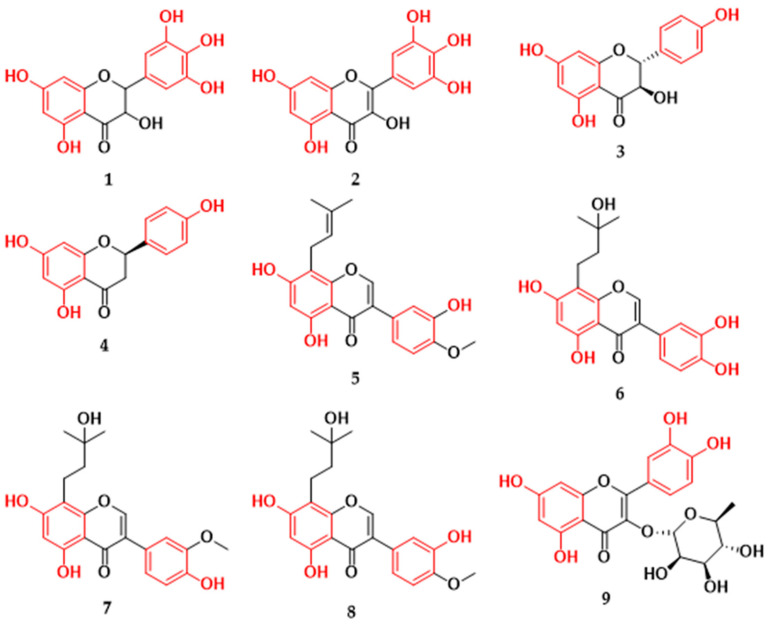

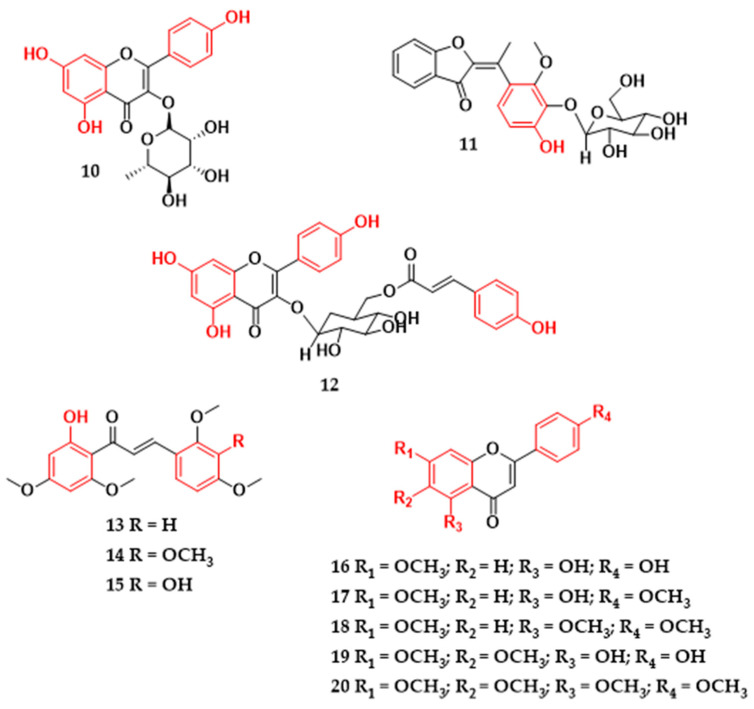
Flavonoids with antibacterial activity isolated from plants in Brazil. Ampelopsin (**1**), myricetin (**2**), dihydrokaempferol (**3**), (+)-(2R)-naringenin (**4**), 3,7,3′-trihydroxy-4′-methoxy-8-prenylisoflavone (**5**), 8-(3-hydroxy-3-methylbutyl)-5,7,3′,4′-tetrahydroxyisoflavone (**6**), 8-(3-hydroxy-3-methylbutyl)-5,7,4′-trihydroxy-3′-methoxyisoflavone (**7**), 8-(3-hydroxy-3-methylbutyl)-5,7,3′-trihydroxy-4′-methoxyisoflavone (**8**), 3-*O*-α-L-rhamnopyranosylquercetin (**9**), 3-*O*-α-L-rhamnopyranosylkaempferol (**10**), (*E*)-3′-*O*-β-d-glucopyranosyl-4,5,6,4′-tetrahydroxy-7,2′-dimethoxyaurone (**11**), tiliroside (**12**), 2′-hydroxy-4,4′,6′-trimethoxychalcone (**13**), 2′-hydroxy-3,4,4′,6′-tetramethoxychalcone (**14**), 3,2′-dihydroxy-4,4′,6′-trimethoxychalcone (**15**), genkwanin (**16**), 7,4′-dimethylapigenin (**17**), trimethylapigenin (**18**), cirsimaritin (**19**), tetramethylscutellarein (**20**).

In the Brazilian territory, there are several reports of isolated substances from the class of flavonoids as potential pathogen inhibitors. We can mention, for example, that, from the roots of *Euphorbia tirucalli* species, two flavonoids were isolated that proved to be potent inhibitors against the bacteria *Escherichia coli* (*E. coli*; ATCC 8739) and *Staphylococcus aureus* (*S. aureus*; ATCC 6538) [16]. This fact is interesting due to the antimicrobial potential of flavonoids being more related to Gram-positive bacteria than Gram-negative bacteria, and the lesser action on Gram-negative bacteria would be due to the presence of negatively charged LPS of the outer bacterial membrane [57]. When we analyzed the molecules tested in Brazil, the flavononol ampelopsin (**1**) and the flavonol myricetin (**2**) (Figure 3) showed higher inhibition values against *S. aureus* (Gram-positive) of 8 μg/mL and 16 μg/mL, respectively [16]. For *E. coli* (Gram-negative), both flavonoids showed antibacterial activity superior to that of the control antibiotic (tetracycline) with a minimum inhibitory concentration of 32 μg/mL, while ampelopsin presented a MIC of 16 μg/mL, and myricetin presented a MIC of 8 μg/mL (Table 1) [16]. An important point is that flavonol myricetin has also been described as a potent multitarget antivirulence agent against the pathogen *S. aureus*, with antibiofilm, antihemolytic, and antistaphyloxanthin properties [60].

In addition, it is suggested that the increase in antibacterial activity against *S. aureus* strains in flavonoids is related to the hydroxylation of carbons at positions C-5, C-7, C-3′, and C4′ [61], which may explain the high inhibitory value for the molecules. The structures of flavonoids (**1**) and (**2**) show the same substitution patterns, with hydroxyl groups on carbons C-3, C-5, C-7, C-3′, C-4′, and C-5′, differing only in the unsaturation of the C ring. In work carried out relating the structure and activity of several flavonoids to the inhibition of *Escherichia coli*, the results showed more efficient inhibitory values in the flavonoids of the flavonol class [10]. Furthermore, some studies indicate that hydroxyl groups at the C-3 position are important for antibacterial activity and contribute to decreasing the cell membrane fluidity of *E. coli* bacteria, which may be one of the direct inhibitory mechanisms of flavonoids. In addition, it was observed that the hydrophobicity and electronic properties of flavonoids can determine their antibacterial activity against *E. coli* [10,52]. Therefore, although there may be a greater propensity for flavonoids to inactivate Gram-positive bacteria, some molecules may be equally effective in inactivating Gram-negative bacteria, ensuring a characteristic of flavonoids as multitarget compounds. Regarding compounds with action on Gram-positive bacteria, the flavonoids dihydroxykaempferol (**3**) and naringenin (**4**) (Figure 3), isolated from the stem of *Maurutia flexuosa* species, were shown to be active against methicillin-susceptible *Staphylococcus aureus* (MSSA) and methicillin-resistant *S. aureus* (MRSA) [17]. Dihydroxykaempferol presented a MIC value of 250 μg/mL for both strains, while the flavanone naringenin showed a more efficient MIC of 62.5 μg/mL (Table 1) [17]. It is important to note that there are works related to the flavonoid naringenin that describe its antibacterial potential against the bacteria *S. aureus* [61]. In a broth dilution assay, naringenin, when combined with an antibiotic, had considerably increased antibacterial activity against multidrug-resistant *S. aureus* strains [62]. In addition, this compound presented antibiofilm properties, and the probable mechanism of action that leads to inhibition of *S. aureus* occurs through the rupture of the bacterium’s cytoplasmic membrane and binding to its genomic DNA [63,64].

Isoflavonoids are another subclass that has been reported to have antibacterial activities in Gram-positive bacteria and are one of the subclasses of flavonoids that are most frequently reported in plants of the *Fabaceae*/*Leguminosae* family [65]. They are structures that differ from flavones by the rearrangement of aromatic ring B in the C-2 to C-3 position through the action of the enzyme dependent on cytochrome P-450 [37]. The greatest structural variation of these compounds occurs by the substituent groups, hydroxyl, methoxyl, methylenedioxy, glycoside, and prenyl groups, which influence the antibacterial activity of these compounds [66]. Studies suggest that prenyl units on carbons at positions C-6, C-8, and C-3′ and hydroxyl groups at positions C-5 and C-7 may contribute to the inhibition of methicillin-resistant *Staphylococcus aureus* strains [67,68]. It is hypothesized that prenyl groups enhance bacterial cell membrane penetration by the attachment of a strongly lipophilic arm to the molecule. Furthermore, diprenylation of isoflavones may be associated with lower minimum inhibitory concentration values against *E. coli* [69]. Isoflavonoids linked to phenyl groups have already been shown to be effective against Gram-positive bacteria such as *S. aureus* and *B. subtilis*, with greater antibacterial activity when the phenyl group is in positions C-6, C-8, C-3′, and C-5′ of the isoflavonoid [70]. Of the isoflavonoids isolated from the leaves of *Vaitera guianensis*, the substances that showed antibacterial activity against methicillin-resistant *S. aureus* were the prenylated isoflavones 5,7,3′-trihydroxy-4′-methoxy-8-prenylisoflavone (**5**), 8-(3-hydroxy-3-methylbutyl)-5,7,3′,4′-tetrahydroxyisoflavone (**6**), 8-(3-hydroxy-3-methylbutyl)-5,7,4′-trihydroxy-3′-methoxyisoflavone (**7**), and 8-(3-hydroxy-3-methylbutyl)-5,7,3′-trihydroxy-4′-methoxyisoflavone (**8**) (Figure 3; Table 1). Compound (**5**) exhibited the highest *S. aureus* inhibition value at the half-maximal inhibitory concentration (IC_50_) of 6.8 µM and was also active against *E. faecium* (IC50 of 12.8 µM) [18]. In work carried out to investigate the effect of soybean flavone on the DNA and RNA of *S. aureus* (ATCC 26112), the authors indicated that isoflavones were effective in inhibiting the activity of topoisomerase I and II enzymes, responsible for the dynamic control of changes in nucleic acids, and their inhibition affected nucleic acid synthesis and bacterial growth [71].

The flavonoid glycosides found in the leaves of the *Clusia burlemarxii* species showed promising antibacterial values. The flavonoid 3-*O*-α-L-rhamnopyranosylquercetin (**9**) (Figure 3) exhibited strong activity against *S. aureus* (ATCC 6538), while the flavonoid 3-*O*-α-L-rhamnopyranosylquercetin (**10**) showed moderate and low activity against *B. subtilis* (ATCC 6633) and *S. aureus* (ATCC 6538), respectively (Table 1) [19]. Both (E)-3′-*O*-β-D-glucopyranosyl-4,5,6,4′-tetrahydroxy-7,2′-dimethoxyaurone (**11**) and tiliroside (12) glycosides from *Gomphrena agrestis* species showed low inhibitory activity against bacterial strains (Table 1) [20]. However, when flavonoid (**12**) was tested in the presence of antibiotics at various concentrations, this flavonoid compound modulated the activity of the antibiotics and reduced the concentration required to inhibit *S. aureus* (AS-1199B). This activity may be related to the lipophilicity attributed to the flavonoid skeleton [21]. Similar results were observed with flavonoid glycosides in inhibiting the pathogen *E. coli* and methicillin-resistant *S. aureus*, and it was indicated that the glycosidic bond influences the low antibacterial activity of the flavonoid [10,72], which may be related to the lack of affinity for the phospholipid layer or to specific receptors in cell membranes [73]. In a comparative study of the flavonoids glucones and aglycones, it was observed that, although the compounds with *O*-glycosides presented no satisfactory values of inhibitory effect on the growth of *S. aureus* strains, this compound had a positive result when the authors evaluated the ability to reduce biofilm complex [74].

Through the phytochemical study of the leaves of *Piper hispidum* species collected in the southern region of Brazil, three flavonoids of the chalcone class were found that expressed antibacterial activity. Chalcones (**13**–**15**) (Figure 3) exhibited MIC values between 125–250 μg/mL for *S. aureus* (ATCC 25923) (Table 1) [22]. Chalcones (1,3-diaryl-2-propen-1-ones) present an open chain α,β-unsaturated flavonoid and are compounds of natural or synthetic origin, occur as cis and trans isomers, and act as precursors for several other natural flavonoid derivatives [37,75,76]. The substituent groups and their respective locations in the molecule interfere with the biological activity of chalcones. The introduction of hydroxyl substituents in the C-2 or C-4 positions of the B ring or C-2′ of the A ring of chalcone can influence the anti-MRSA activity; on the other hand, methoxyl groups linked to the structure prevent staphylococcal activity. It is very common for chalcones of natural origin to have a hydroxyl group at the C-2′ position, which helps stabilize the molecule. However, this presence may not be fundamental in its antibacterial activity [13,77,78]. Furthermore, studies suggest that only one hydroxyl group on the B ring of chalcones may not be sufficient to exhibit significant activity in inhibiting *S. aureus* [79]. In contrast, antibacterial activity against Gram-positive bacteria *S. aureus* and *B. cereus* may be related to the presence of an oxygenated substituent group on the C-4′ carbon, hydroxyl on the C-4 carbon, and an isoprenoid side chain, located on the C-3′ carbon [13].

In the last decade, the emergence of antibiotic-resistant strains has increased alarmingly [80]. As a result, the emergence of multiresistant bacteria occurs, leading to drug ineffectiveness, prolonging the duration of the disease, and leading to death [81]. A strategy to combat bacterial resistance is the use of compounds that can suppress cellular resistance mechanisms, increasing the effectiveness of existing antibiotics [82,83]. There are reports of compounds from the class of flavonoids that, when combined with drugs, potentiate the inhibitory effect of the drug [84,85,86,87]. As an example, the natural compounds genkwanin (**16**), 7,4′-dimethylapigenin (**17**), trimethylapigenin (**18**), cirsimaritin (**19**), and tetramethylscutellarein (**20**) (Figure 3), isolated from the aerial parts of *Praxelis clematidea* species, did not show significant results against *S. aureus* (SA-1199B0) when tested alone. However, these compounds proved to be good modulators of the activity of the antibiotics norfloxacin and ethidium bromide. In particular, compound (**20**) was able to reduce the MIC of drugs against pathogens by sixteen-fold (Table 1). This property may be related to the position of the methoxy groups in the 4′, 5, 6, and 7 positions of the flavone, which increases its lipophilicity [12]. In general, lipophilicity is an important factor in flavonoids for the inhibition of Gram-positive bacteria [88]. It is influenced by the pH and structural characteristics of the compounds (substituent groups and their respective locations in the backbone of the structure). It has been seen that hydroxyl groups and isopentenyl substituents on the A, B, and C rings of the flavonoid influence the lipophilicity of flavonoids and may be more effective in antibacterial activity [88].

## 5. Xanthones

Xanthones (derived from the Greek “yellow”) are symmetric oxygenated heterocyclic derivatives with a basic skeleton of dibenzo-γ-pyrone [89,90]. They are polyphenolic secondary metabolites found in fungi, lichens, bacteria, and mainly in plants from the families *Gentianaceae*, *Polygalaceae*, *Clusiaceae*, and *Moraceae* [91,92]. They are divided into five groups: simple xanthones, glycosides, xanthonolignoids (frequent in the *Gentianaceae* family), prenylated xanthones (more frequent in the *Clusiaceae* family), and the xanthones of the miscellaneous group, which have unusual substitutions and are extracted from different plant and vegetable sources [93].

**Figure 4:**
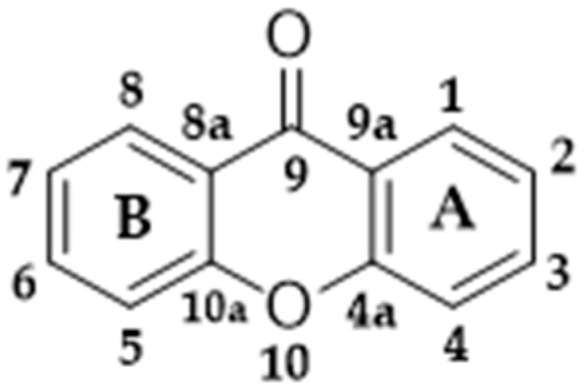
Basic structure of xanthones.

When produced by higher plants, xanthones are formed by a mixed biosynthetic pathway (shikimate-acetate) [93]. The phenylalanine produced in the shikimate pathway is oxidized to m-hydroxybenzoic acid, which reacts with three acetate units, forming the intermediate benzophenone, and through intramolecular reactions, xanthones are formed [92,94]. Molecular numbering occurs according to a biosynthetic convention. The carbons of the A ring (acetate pathway) of xanthones are numbered 1–4, while the B ring (shikimate pathway) is numbered 5–8 (Figure 4) [95].

There are many reports on numerous pharmacological activities attributed to xanthones of natural origin, including anti-inflammatory [96,97], anti-Leptospira [98], anti-collagenase, anti-elastase, anti-hyaluronidase, anti-tyrosinase [99], anticancer [100,101], antidiabetic [102,103], and antifungal [104] activities, and many xanthones have been described as good bacterial inhibitors [105,106,107,108].

**Figure 5:**
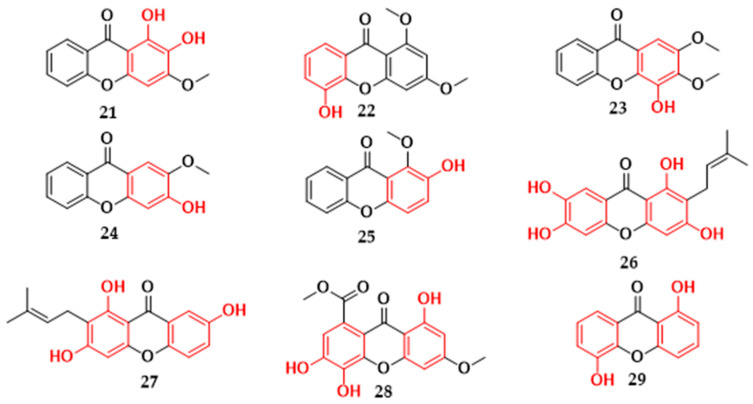
Xanthones with antibacterial activity isolated from plants in Brazil. 3,4-dihydroxy-2-methoxyxanthone (**21**), 5-hydroxy-1,3-dimethoxyxanthone (**22**), 4-hydroxy-2,3-dimethoxyxanthone (**23**), 3-hydroxy-2-methoxyxanthone (**24**), 2-hydroxy-1-methoxyxanthone (**25**), Assiguxanthone B (**26**), 1,3,7′trihydroxy-2-(3-methylbut-2-enyl)-xanthone (**27**), 8-carboxymethyl-1,3,5,6-tetrahydroxyxanthone (**28**), 1,5-dihydroxyxantona (**29**).

In a phytochemical investigation with *Kielmeyera variabilis* species collected in southeastern Brazil, oxygenated xanthones with a potential inhibitor of multidrug-resistant strains of *S. aureus* were isolated. The xanthones (**21**–**25**) (Figure 5) found in the leaves of this species exhibited MIC values ranging between 16–128 mg/L (Table 1). Among the compounds, xanthone 3,4-dihydroxy-2-methoxyxanthone (**21**), structurally differentiated by having a catechol group, exhibited greater antibacterial activity [23]. From the stems of the same plant species, the isolation of prenylated xanthone assiguxanthone B (**26**) (Figure 5) was reported, which proved efficient against *S. aureus* (ATCC 25923) and *B. subtilis* (ATCC 6623), with MICs of 100 μg/mL and 25 μg/mL, respectively (Table 1) [24].

The prenylated xanthone 1,3,7′trihydroxy-2-(3-methylbut-2-enyl)-xanthone (**27**) (Figure 5) isolated from *Kielmeyera coriacea* species proved to be a potent inhibitor against the Gram-positive bacteria *S. aureus* (ATCC 25923) and *B. subtilis* (ATCC 6623), exhibiting a MIC value of 12.5 μg/mL (Table 1) [25]. From the leaves of *Leithrix spiralis* species, the compound 8-carboxymethyl-1,3,5,6-tetrahydroxyxanthone (**28**) (Figure 5) was reported, showing inhibition values of 125 μg/mL against *S. aureus* (ATCC 25923), *B. subtilis* (ATCC 19659), and *P. aeruginosa* (ATCC 27853) (Table 1) [26]. In *Calophyllum brasiliense*, oxygenated xanthone 1,5-dihydroxyxanthone (**29**) (Figure 5) showed moderate activity against the strains *S. aureus*, *S. saprophyticus*, and *S. agalactiae* and weak activity against *B. cereus* (Table 1) [27].

The substitution pattern in xanthone molecules is of high importance to understand their antimicrobial activity. Studies that relate structure and activity indicate that hydroxyl groups at the C-3 and C-6 positions and side chain prenyl groups at the C-2 carbon play a prominent antibacterial role against MRSA [11,109], as seen in compound (**26**). Prenylated xanthones have great antimicrobial potential, as the existence of apolar groups increases membrane permeability and can act as modulators of lipid affinity and cellular bioavailability [23]. However, a decrease in the anti-MRSA activity of synthetic xanthones modified with diethylamine groups was verified when long nonpolar alkyl chains were introduced in the structure, which confers hydrophobic characteristics to the structure and would have made it difficult to penetrate the peptidoglycan layer of the bacteria [110].

Methoxyl substituents at positions C-3 and C-7 and isoprenyl at positions C-2 and C-8 seem to contribute to better activity against Gram-positive bacteria, while geraniol and isoprenyl groups at positions C-4 and C-2 may contribute to a higher antibacterial activity against *P. aeruginosa*. However, bulky groups at the C-1 position may decrease the antibacterial activity [111]. In methicillin-resistant *S. aureus* strains, xanthones with hydroxyls at positions C-6, C-5, and C7, prenyl groups at C-4 and C-7, and dimethyl chromene rings at C-2 and C-3 present efficient activity [112]. Furthermore, Yan et al. [113], in a study evaluating xanthones with various substituents at the C-1, C-3, and C-6 positions, found that an acetyl substituent group at the C-1 position showed membrane selectivity against *S. aureus*, a higher inhibition of biofilm formation, and better antibacterial activity in vivo when compared to other xanthones [113].

Durães et al. [114] evaluated the potential of xanthones as efflux pump inhibitors in *Staphylococcus aureus* (272123) and *Salmonella enterica* serovar Typhimurium (SL1344) strains. It was observed that the introduction of hydroxyl groups in each aromatic ring at positions C-1 and C-7, or in the same plane as the ketone functional group of xanthone, as well as the presence of bulky groups at position C-1, was demonstrated to be efficient in inhibiting *Salmonella enterica*. For *Staphylococcus aureus*, methoxyl groups at the C-6 position can exert an important influence on the inhibition of the efflux pump; however, bulky groups at C-1 seem to impair the activity [114].

Similar to flavonoids, the mechanism of action of xanthones is also related to the ability to partition the bacterial cell membrane and has a greater relationship with Gram-positive bacteria. An important point is that xanthones have recently been widely explored due to the wide range of different molecular substitutions that can modulate several biological responses, such as those mentioned above [115]. This is because, in addition to the multiple functions of xanthones, natural structures can serve as inspiration for the synthesis of new xanthones with diverse molecular functions. Therefore, the molecules discussed below have the potential for use in natura or may serve as a basis for future synthetic derivations.

## 6. Coumarins

Coumarins are chemical compounds of natural or synthetic origin; they are stable and of low molecular weight, called benzo-α-pyrone, and have the isomeric chemical structure of chromones (benzo-γ-pyrone). As natural products, they can be produced by fungi and bacteria and in the secondary metabolism of plants [116,117]. The first report of isolation occurred in 1820 from the seeds of *Dipteryx odorata* species, popularly known as tonka bean, by A. Vogel, a regular member of the Royal Academy of Science in Munich [118]. Currently, the isolation of coumarins in hundreds of plant species is already known, with greater occurrence in the families *Apiaceae*, *Rutaceae*, *Asteraceae*, *Fabaceae*, *Oleaceae*, *Moraceae*, and *Thymelaceacea* [119]. Although coumarins are reported to occur in all parts of plants, they are most commonly found in fruits, roots, stems, and leaves [120].

Coumarins are compounds derived from the ortho-hydroxylation reaction of cinnamic acid, forming 2-coumaric acid, which undergoes cis–trans isomerization followed by lactonization, giving rise to the base structure of coumarin [37,120]. Coumarins can be divided into four major subgroups, which include single coumarins, pyrone-substituted coumarins, furanocoumarins, and pyranocoumarins [121]. From a pharmacological point of view, coumarin compounds and their derivatives are of great importance in the prevention and treatment of diseases. Some are used as anticoagulants, antitumor agents, antispasmodics, choleretic drugs, and antibiotics, such as the drug novobiocin, a potent inhibitor of Gram-positive bacteria [122,123].

**Figure 6:**
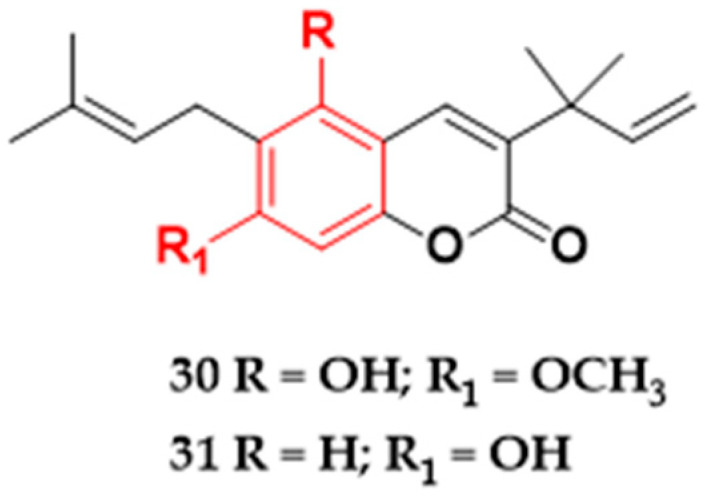
Coumarins with antibacterial activity isolated from plants in Brazil. Tanizin (**30**), and gravellifenore (**31**).

The coumarins tanizin (**30**) and gravellifenore (**31**) (Figure 6) are examples of coumarin compounds with good inhibitory potential against bacterial strains. Isolated from the bark of *Helietta apiculata* species, both compounds showed MIC values ≤ 50 μg/mL against Gram-positive and Gram-negative bacteria (Table 1) [28]. Structurally, the two substances differ only in the C-5 and C-7 carbon positions, which may be reflected in the different MIC values seen in Table 1. Studies indicate that the coumarin substitution patterns are related to their pharmacological and biochemical activities and therapeutic applications [119].

In coumarins, it was seen that the addition of polar or nonpolar groups and their respective locations can interfere with the antibacterial activity. The introduction of a hydroxyl group at the C-7 position of the aromatic ring can reduce the antibacterial activity. However, the addition of two methoxyl groups at the C-7 and C-8 positions can make the compounds more active against Gram-positive and Gram-negative microorganisms [124]. Other studies have revealed that, in mono-oxygenated coumarins, the addition of methoxyl or methyl groups at the C-6 and C-7 positions may decrease the antibacterial activity against Gram-positive strains. It is suggested that the lipophilic character and planar structure of coumarins are factors that may confer reasonably high antibacterial activities on these structures. [125]. In a preliminary study of the structure–activity relationship, it was shown that the inclusion of biphenyl groups at the C-3 position can confer strong activity against the DNA helicases of Gram-positive bacteria. However, when ester functionality was tested at this position, inactive compounds were found. Furthermore, it was seen that the change of substituents at the C-7 position influences the effectiveness against both DNA helicases [126]. Additionally, some coumarins are potential efflux pump inhibitors. In a study with seven coumarins, it was observed that phenyl groups at C-4,2-methylbutanoyl at C-6 and prenyl at C-8 seem to contribute to the inhibition of the efflux pump in the clinical strain *S. aureus* 1199B [127].

## 7. Phenolic Acids

Phenolic acids (phenolcarboxylic acids) are ubiquitous compounds in plants and are frequently reported in fruits, vegetables, spices, and herbs, in addition to being found in fungi and bacteria. In food, phenolic acids are associated with nutritional, antioxidant, and organoleptic properties, while in the survival mechanism of plants, these compounds contribute to protein synthesis, photosynthesis, nutrient absorption, and allelopathy [128,129]. Several phenolic acids have been described to have many biological activities, including antifungal, antioxidant, antibacterial, and anti-inflammatory activities [130,131,132,133,134,135,136,137,138].

Structurally, phenolic acids are characterized by having an aromatic ring directly linked to a hydroxyl group (phenolic hydroxyl) and a carboxyl group and can be found in nature conjugated to esters, ethers, simple sugars, vegetable polymers, organic acids, or polyphenols [139,140]. They are compounds produced in greater numbers in the shikimic acid pathway through the precursors L-phenylalanine or L-tyrosine. These phenolic compounds have two distinct structures: hydroxycinnamic and hydroxybenzoic. Hydroxycinnamic acids or cinnamic acids (C6-C3) can be produced by all plants through the deamination of L-phenylalanine; however, the formation of cinnamic acids from L-tyrosine is restricted to some plants. Hydroxybenzoics (C6-C1), on the other hand, can be formed at the beginning of the shikimate pathway through intermediates and through alternative routes by derivatives of hydroxycinnamic acids [37,141,142,143]. Similar to other compounds previously discussed in the present study, phenolic acids affect bacteria through damage to the cell membrane wall. This effect leads to a change in cell surface hydrophobicity and charge, with consequent leakage of cytoplasmic content [144]. In a study of the antibacterial mechanism using rosmarinic acid, inhibition of Gram-positive and Gram-negative bacteria was estimated by the destruction of bacterial cells and cellular proteins in addition to inhibition of Na+/K+-ATP-ase activity [145]. However, although the mechanism of action on the cell wall of bacteria has been elucidated, as well as coumarins, phenolic acids can also act on fungi through the interaction between caffeic acid derivatives and the 1,3-β-glucan synthase fraction [146].

**Figure 7:**
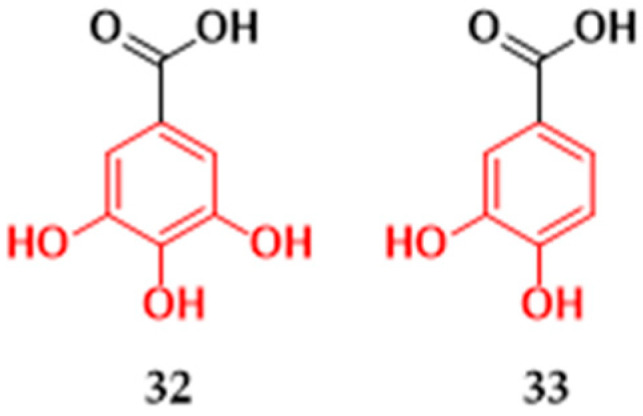
Phenolic acids with antibacterial activity isolated from plants in Brazil. Gallic acid (**32**) and protocatechuic acid (**33**).

Gallic (**32**) and protocatechuic (**33**) acids (Figure 7) were tested for their antibacterial activities against Gram-positive and Gram-negative strains. Gallic acid isolated from *Himatanthus sucuuba* species in northern Brazil showed inhibitory activity against *S. aureus* (MRSA), *S. epidermidis*, *P. mirabilis*, *S. haemolyticus*, and *E. coli*, with values ranging between 31 and 125 μg/mL (Table 1). The highest efficacy values were against methicillin-resistant *S. aureus* and *P. mirabilis* [29]. The protocatechuic acid isolated from the aerial parts of *Calophyllum brasiliense* differs from gallic acid only by the absence of hydroxyl in the C-5 position of the aromatic ring, showing MIC against all tested strains with values from 200 to 700 μg/mL (Table 1). In the same study, gallic acid was tested against the same bacterial strains but did not show activity against the tested microorganisms up to a concentration of 1000 μg/mL [27].

As with other phenolic compounds, the antimicrobial activity of phenolic acids depends on the chemical structure of the compounds. It has been seen that the number and positions of the substituent groups on the aromatic ring, the length of the side chain, and the unsaturations influence the increase or decrease of the antibacterial activity of phenolic acids [147]. For example, in studies of the inhibitory action of caffeic acid and caffeic acid alkyl esters against *S. aureus* and *E. coli*, it was observed that compounds with long alkyl side chains were more efficient in inhibiting Gram-positive bacteria, and medium alkyl chains were more potent in inhibiting Gram-negative bacteria [148].

The lipophilicity of phenolic acids is one of the determining factors for the antimicrobial potential; the greater the lipophilicity of these compounds, the greater the inhibitory capacity. Studies have observed that as hydroxyl groups are replaced by methoxyl, there is unsaturation in the molecules, in addition to a decrease in pH, which causes acidification in the plasmatic membrane of pathogens; thus, there is an increase in the lipophilicity of phenolic acids, consequently improving the antibacterial activity [14,15].

## 8. Other Phenolic Compounds

**Figure 8:**
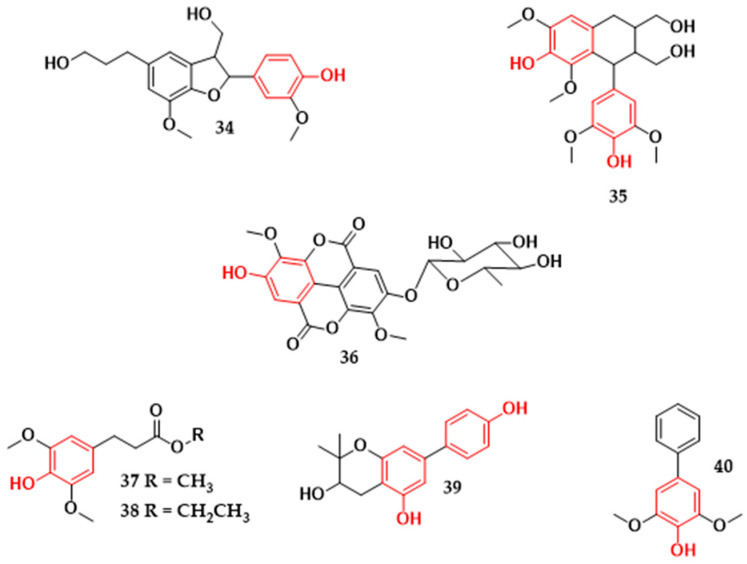
Other classes of phenolic compounds with antibacterial activity isolated from plants in Brazil. Dihydrodehydrodiconiferyl alcohol (**34**), lyoniresinol (**35**), Acid 3,3′-dimethoxyellagic acid-4-*O-*α-rhamnopyranoside (**36**), (E)-methyl-4-hydroxy-3,5-dimethoxycinnamate (**37**), (E)-ethyl-4-hydroxy-3,5-dimethoxycinnamate (**38**), 2,2-dimethyl-3,5-dihydroxy-7-(4-hydroxyphenyl) chromane (**39**), aucuparin (**40**).

Other types of phenolic compounds were also found in plants from Brazil and tested for their inhibitory action against microorganisms. The neolignan, dihydro-dehydrodiconiferyl alcohol (**34**), and the lignan, Lyoniresinol (**35**) (Figure 8), were active against *S. aureus* strains. Compound (**34**), isolated from *Styrax ferrugineus* species, showed potent inhibitory action against *S. aureus* ATCC 12228 (MIC 20 μg/mL) [30]. Lignan (**35**) from *Clusia burlemarxii* leaves showed a MIC value of 25 μg/mL against the *S. aureus* strain (ATCC 6538) [19]. Lignans and neolignans are natural products formed by two phenylpropane units (C6C3) through oxidative dimerization [149,150]. When the formation of the molecule occurs by the β, β’ bond, the term used is lignan; in the absence of this bond, the molecule formed by two C6-C3 units is neolignan [151]. Regarding the structure activities of the lignans, the substituents and the absolute configuration in the hydrofuran rings seem to affect the antibacterial activity in these compounds, and the presence of methoxyl groups in the C-9 and C-9′ positions can cause the inactivation of the compound against Gram-positive bacteria [152]. Although there are lignans that are reported to have antibacterial activity [153,154,155,156], studies relating to the structure-activity of these compounds are scarce.

The ellagic acid derivative (**36**) (Figure 8) isolated from the roots of *Euphorbia tirucalli* exhibited inhibitory activity against *S. aureus* (ATCC 6538) and *E. coli* (ATCC 8739), being more active against *S. aureus* (Table 1) [16]. The cinnamic acid derivatives (E)-methyl-4-hydroxy-3,5-dimethoxycinnamate (**37**) and (E)-ethyl-4-hydroxy-3,5-dimethoxycinnamate (**38**) (Figure 8) from the bark of *Hellietta apiculata* species were active against Gram-positive and Gram-negative bacteria (Table 1). In particular, compound (**37**) showed a greater inhibitory effect than compound (**38**) against *B. cereus* (ATCC 33019) and *E. aerogenes* (ATCC 13048), with a minimum inhibitory concentration value up to four-times lower [28]. Compounds (**37**) and (**38**) structurally differ only in the methyl and ethyl substituents of the side chain of the compound.

Biphenyl 2,2-dimethyl-3,5-dihydroxy-7-(4-hydroxyphenyl) chromane was reported from *Clusia burlemarxii* species (**39**) (Figure 8) and showed antibacterial activity against four pathogens (Table 1). The higher activity was against the microorganisms *M. luteus* ATCC 10240 (MIC = 25 μg/mL) and *S. aureus* ATCC 6538 (MIC = 50 μg/mL) [19]. Another biphenyl, known as aucuparin (**40**) (Figure 8), isolated from *Kielmeyera coriacea* has shown antibacterial activity against *S. aureus* (ATCC 25922), *E. coli* (ATCC 25922), *B. subtilis* (ATCC 6623), and *P. aeruginosa* (ATCC 15442) (Table 1). It is more active against Gram-positive bacteria, with minimum inhibitory concentrations of 3.12 μg/mL and 12.5 μg/mL against *B. subtilis* and *S. aureus*, respectively [25]. Studies indicate that the formation of biphenyl aucuparin in plants occurs in response to attacks by microorganisms [157].

## 9. Phenolic Compounds and a Possible Farm-to-Fork Influence

The use of natural compounds comprises an important strategy in the control of pathogens, from the farm to the health of the consumer. Studies have evaluated that, in cattle fed a forage-based diet (containing a high presence of phenolic compounds), there was less elimination of *E. coli* O157:H7 in the animals’ feces [158]. The load of *E. coli* O157:H7 shed in bovine feces is of special importance since super-shedding events have already been reported and are responsible for the high presence of this pathogen in animal feces [159]. The super-shedding event was related to the presence of 80% hide contamination in all animals present in a feedlot [160]. Thus, the action of phenolic compounds has the potential to be explored in animal feed to reduce the concentration of bacteria in feces, which consequently could reduce contamination in animal hide and its prevalence in the industry.

In addition, the application of phenolic compounds in food production has been extensively discussed recently. In a study performed by Zamuz et al. [161], the authors mentioned the potential of using phenolic compounds in the fight against *Listeria monocytogenes*, an important human pathogen that presents characteristics of microbial growth under refrigeration and potential for biofilm formation. The production of microbial biofilms in the food industry represents one of the main contamination factors during the food process [162]. This microbial strategy allows the formation of layers of cells that help in the persistence of the microorganism during food production, since the application of sanitizers will act on the outermost layers, protecting the deeper layers of microorganisms from the action of the substance [163]. In several studies, flavonoids, such as naringin, were indicated as agents with the potential to be nonspecific inhibitors of autoinducer-mediated cell–cell signaling in *E. coli* bacteria [164]. This interference can have a direct impact on bacterial biofilm formation since it is estimated that communication between bacterial cells plays a crucial role in biofilm formation. Nevertheless, other studies have identified the ability of flavans to inhibit the formation of *S. aureus* biofilms, both with natural molecules and with synthetic forms [165]. This fact allows the substance to be obtained in larger quantities to enable large-scale application. Additionally, the presence of microbial biofilms can lead to resistance to these compounds, since subinhibitory amounts of sanitizer could lead to selective pressure on the isolates and the consequent development of resistance mechanisms [166]. As previously mentioned, the use of synthetic xanthones to combat multidrug resistance has already been addressed by Duraes et al. [114]. The authors highlight the role of seven xanthone compounds in decreasing the efflux of ethidium bromide, which can be translated as an efflux pump inhibitor [114]. Drug efflux is the main mechanism of resistance in Gram-negative bacteria [167]. This allows the bacteria to regulate the cell’s internal contents and enable the removal of toxic substances, including antimicrobial compounds [168]. Thus, the use of phenolic compounds could be another ally in combating biofilm contamination and antimicrobial resistance.

Remaining in food production, the use of phenolic compounds can be a good strategy to extend the shelf life of a product, acting as an active component in the packaging [169]. In a study performed by Gaikwad [170], the authors verified that the use of pyrogallol coated in a polymeric film obtained an antimicrobial effect in both Gram-positive (*S. aureus*) and Gram-negative (*E. coli*) bacteria. Such results highlight the power of phenolic compounds since the molecule affected different bacterial groups. An important point to be emphasized in the use of phenolic compounds in packaging is that these substances, in addition to their antimicrobial action, also have an antioxidant effect, which contributes to increasing the shelf life and maintaining the chemical quality of the product. In a study performed by Kalogianni [171], the author discussed important aspects of the use of phenolic compounds in the meat industry, such as the direct application of the compounds in the meat and inclusion in the animal diet.

In addition to animal and food production, the use of phenolic compounds through diet has been widely studied in human health. Although most studies have focused on the antioxidant properties of these substances, some studies have aimed to investigate the role of phenolic compounds in the gut microbiota. It is important to note that in recent years, several studies have linked diseases with the intestinal microbiota through dysbiosis [172], and a diet composed of polyphenols could be a key factor in helping this problem, since phenolic compounds are also related to the expression of prebiotic properties, leading to antimicrobial activity against microbial pathogens [172]. Furthermore, in a study performed by Tuohu et al. [173], the authors pointed out that, although the fundamentals about the role of polyphenols are not clear in the gut microbiota, the anti-age, antimicrobial, and anti-inflammatory properties point to a potential benefit when consumed in adequate amounts. The benefits of a polyphenol-based diet have been evaluated in vivo; for example, in the study performed in Brazil by Gris et al. [174], the authors verified that the administration of red wines with a high phenolic content promoted high antioxidant activity in mice. Furthermore, in another study in the country, the authors highlighted that extracts from murici and gabiroba (Brazilian Cerrado fruits) obtained protective effects against genotoxic and mutagenic inducers in mice [175]. In addition, in a study performed by Siqueira et al. [176], the authors verified that extracts derived from *Spondias tuberosa* (a fruit from a plant native to northeast Brazil) presented the molecules of chlorogenic acid, caffeic acid, rutin, and isoquercitrin that showed therapeutic potential in inflammatory conditions in mice. Therefore, as mentioned throughout the text, phenolic compounds have wide potential for use, from the farm in animal production to the quality of human health, and represent a strong ally in the search for natural compounds that help in the fight against microbial contamination and, at the same time, help in other activities such as cellular antioxidation and anti-inflammatory properties.

## 10. Conclusions and Future Perspectives

As discussed throughout the manuscript, there are several phenolic compounds with different properties, such as antimicrobial, antiaging, and anti-inflammatory properties. In the present study, we discuss substances isolated from plants in Brazil, with a focus on their antimicrobial capacity. We emphasize that, although several studies showed promising values of microbial inactivation, the studies are still in the initial stage “in vitro”, and their application on a large scale is still a limiting factor in the application of these compounds. Furthermore, several studies have analyzed the properties of polyphenols in *Staphylococcus aureus* and *Escherichia coli*, and, although these two microorganisms represent two distinct classes (Gram-positive and negative, respectively), several other pathogens still need to be evaluated to test the effectiveness of the compounds, as well as a possible cross effect in a bacterial community. However, the results presented shed light on promising compounds that can help in food production and human health in the coming years, as we are experiencing the era of multiresistant microorganisms, which, in addition to costing countless lives, also has a high global economic cost [177].

## Figures and Tables

**Table 1:** Phenolic Compounds isolated from plants in Brazil and their respective antibacterial activities.

Structure	Phenolic Compound	Plant	Bacteria	Inhibition	Location	Reference
**Flavonoids**						
**1**	Ampelopsin	*Euphorbia tirucalli*	*S. aureus* (ATCC 6538)	** MIC = 8 μg/mL	Araruna—PB *	[16]
			*E. coli* (ATCC 8739)	MIC = 16 μg/mL		
**2**	Myricetin	*Euphorbia tirucalli*	*S. aureus* (ATCC 6538)	MIC = 16 μg/mL	Araruna—PB	[16]
			*E. coli* (ATCC 8739)	MIC = 8 μg/mL		
**3**	Dihydrokaempferol	*Mauritia flexuosa*	*S. aureus* (ATCC 29213)	MIC = 250 μg/mL	Chapada Gaúcha—MG *	[17]
			*S. aureus* (clinical isolate 155)	MIC = 250 μg/mL		
**4**	(+)-(2R)-naringenin	*Mauritia flexuosa*	*S. aureus* (ATCC 29213)	MIC = 62.5 μg/mL	Chapada Gaúcha—MG	[17]
			*S. aureus* (clinical isolate 155)	MIC = 62.5 μg/mL		
**5**	3,7,3′-trihydroxy-4′-methoxy-8-prenylisoflavone	*Vatairea guianensis*	*S. aureus* (MRSA)	*** IC_50_ = 6.8 μM	Santana—AP *	[18]
			*E. faecium*	IC_50_ = 12.8 μM		
**6**	8-(3-hydroxy-3-methylbutyl)-5,7,3′,4′-tetrahydroxyisoflavone	*Vatairea guianensis*	*S. aureus* (MRSA)	IC_50_ = 29.6 μM	Santana—AP	[18]
**7**	8-(3-hydroxy-3-methylbutyl)-5,7,4′-trihydroxy-3′-methoxyisoflavone	*Vatairea guianensis*	*S. aureus* (MRSA)	IC_50_ = 37 μM	Santana—AP	[18]
			*E. faecium*	IC_50_ = 80.6 μM		
**8**	8-(3-hydroxy-3-methylbutyl)-5,7,3′-trihydroxy-4′-methoxyisoflavone	*Vatairea guianensis*	*S. aureus* (MRSA)	IC_50_ = 49.0 μM	Santana—AP	[18]
**9**	3-O-α-L-rhamnopyranosylquercetin	*Clusia burlemarxii*	*B. subtilis* (ATCC 6633)	MIC = 50 μg/mL	Mucugê—BA *	[19]
			*S. aureus* (ATCC 6538)	MIC = 100 μg/mL		
**10**	3-O-α-L-rhamnopyranosylkaempferol	*Clusia burlemarxii*	*S. aureus* (ATCC 6538)	MIC = 25 μg/mL	Mucugê—BA	[19]
**11**	(*E*)-3′-*O*-β-d-glucopyranosyl-4,5,6,4′-tetrahydroxy-7,2′-dimethoxyaurone	*Gomphrena agrestis*	*S. epidermidis* (6epi)	MIC = 0.1 mg/mL	Alto Paraíso—GO *	[20]
			*S. epidermidis* (epiC)	MIC = 0.5 mg/mL		
			*P. aeruginosa* (ATCC 27853)	MIC = 0.5 mg/mL		
			*P. aeruginosa* (290D)	MIC = 0.5 mg/mL		
**12**	Tiliroside	*Gomphrena agrestis* *Herissantia tiubae*	*S. aureus* (ATCC 25923) *S. aureus* (SA-1199B)	MIC = 0.5 mg/mL MIC = 256 μg/mL	Alto Paraíso—GO Juazeirinho—PB *	[20,21]
**13**	2′-hydroxy-4,4′,6′-trimethoxychalcone	*Piper hispidum*	*S. aureus* (ATCC 25923)	MIC = 125 μg/mL	Maringá—PR *	[22]
**14**	2′-hydroxy-3,4,4′,6′-tetramethoxychalcone	*Piper hispidum*	*S. aureus* (ATCC 25923)	MIC = 250 μg/mL	Maringá—PR	[22]
**15**	3,2′-dihydroxy-4,4′,6′-trimethoxychalcone	*Piper hispidum*	*S. aureus* (ATCC 25923)	MIC = 125 μg/mL	Maringá—PR	[22]
**16**	Genkwanin	*Praxelis clematidea*	*S. aureus* (AS-1199B)	MIC = 64 μg/mL **** (Substance with Norfloxacin) MIC = 128 μg/mL (Nofloxacin)	Santa Rita—PB	[12]
				MIC = 16 μg/mL (Substance with Ethidium bromide) MIC = 32 μg/mL (Ethidium bromide)		
**17**	7,4′-dimethylapigenin	*Praxelis clematidea*	*S. aureus* (AS-1199B)	MIC = 64 μg/mL (Substance with Norfloxacin) MIC = 128 μg/mL (Norfloxacin)	Santa Rita—PB	[12]
				MIC = 16 μg/mL (Substance with Ethidium bromide) MIC = 32 μg/mL (Ethidium bromide)		
**18**	trimethylapigenin	*Praxelis clematidea*	*S. aureus* (AS-1199B)	MIC = 16 μg/mL (Substance with Norfloxacin) MIC = 128 μg/mL (Norfloxacin)	Santa Rita—PB	[12]
				MIC = 8 μg/mL (Substance with Ethidium bromide) MIC = 32 μg/mL (Ethidium bromide)		
**19**	cirsimaritin	*Praxelis clematidea*	*S. aureus* (AS-1199B)	MIC = 32 μg/mL (Substance with Norfloxacin) MIC = 128 μg/mL (Norfloxacin)	Santa Rita—PB	[12]
				MIC = 8 μg/mL (Substance with Ethidium bromide) MIC = 32 μg/mL (Ethidium bromide)		
**20**	tetramethylscutellarein	*Praxelis clematidea*	*S. aureus* (AS-1199B)	MIC = 8 μg/mL (Substance with Norfloxacin) MIC = 128 μg/mL (Norfloxacin)	Santa Rita—PB	[12]
				MIC = 2 μg/mL (Substance with Ethidium bromide) MIC = 32 μg/mL (Ethidium bromide)		
**Xanthones**						
**21**	3,4-dihydroxy-2-methoxyxanthone	*Kielmeyera variabilis*	*S. aureus* (SA-1199B)	MIC = 32 mg/L	Mogi Guaçu—SP *	[23]
			*S. aureus* (XU212)	MIC = 32–16 mg/L		
			*S. aureus* (ATCC 25923)	MIC = 64 mg/L		
			*S. aureus* (RN4220)	MIC = 32 mg/L		
			*S. aureus* (EMRSA-15)	MIC = 64 mg/L		
			*S. aureus* (EMRSA-16)	MIC = 16 mg/L		
**22**	5-hydroxy-1,3-dimethoxyxanthone	*Kielmeyera variabilis*	*S. aureus* (SA-1199B)	MIC = 128–64 mg/L	Mogi Guaçu—SP	[23]
			*S. aureus* (ATCC 25923)	MIC = 128 mg/L		
			*S. aureus* (EMRSA-16)	MIC = 64 mg/L		
**23**	4-hydroxy-2,3-dimethoxyxanthone	*Kielmeyera variabilis*	*S. aureus* (SA-1199B)	MIC = 128–64 mg/L	Mogi Guaçu—SP	[23]
			*S. aureus* (XU212)	MIC = MIC = 128 mg/L		
			*S. aureus* (EMRSA-16)	MIC = 64 mg/L		
**24**	3-hydroxy-2-methoxyxanthone	*Kielmeyera variabilis*	*S. aureus* (SA-1199B)	MIC = 64 mg/L	Mogi Guaçu—SP	[23]
			*S. aureus* (XU212)	MIC = 64 mg/L		
			*S. aureus* (ATCC 25923)	MIC = 64 mg/L		
			*S. aureus* (RN4220)	MIC = 64 mg/L		
			*S. aureus* (EMRSA-15)	MIC = 64 mg/L		
			*S. aureus* (EMRSA-16)	MIC = 32 mg/L		
**25**	2-hydroxy-1-methoxyxanthone	*Kielmeyera variabilis*	*S. aureus* (SA-1199B)	MIC = 64 mg/L	Mogi Guaçu—SP	[23]
			*S. aureus* (XU212)	MIC = 128 mg/L		
			*S. aureus* (ATCC 25923)	MIC = 64 mg/L		
			*S. aureus* (RN4220)	MIC = 64 mg/L		
			*S. aureus* (EMRSA-15)	MIC = 64 mg/L		
			*S. aureus* (EMRSA-16)	MIC = 32 mg/L		
**26**	Assiguxanthone B	*Kielmeyera variabilis*	*S. aureus* (ATCC 25923)	MIC = 100 μg/mL	Mogi Guaçu—SP	[24]
			*B. subtilis* (ATCC 6623)	MIC = 25 μg/mL		
**27**	1,3,7’trihydroxy-2-(3-methylbut-2-enyl)-xanthone	*Kielmeyera coriacea*	*S. aureus* (ATCC 25922)	MIC = 12.5 μg/mL	Mogi Guaçu—SP	[25]
			*E. coli* (ATCC 25922)	MIC > 100 μg/mL		
			*B. subtilis* (ATCC 6623)	MIC = 12.5 μg/mL		
			*P. aeruginosa* (ATCC 15442)	MIC > 100 μg/mL		
**28**	8-carboxymethyl-1,3,5,6-tetrahydroxyxanthone	*Leiothrix spiralis*	*S. aureus* (ATCC 25923)	MIC = 125 μg/mL	Diamantina—MG	[26]
			*B. subtilis* (ATCC 19659)	MIC = 125 μg/mL		
			*P. aeruginosa* (ATCC 27853)	MIC = 125 μg/mL		
**29**	1,5-dihydroxyxanthone	*Calophyllum brasiliense*	*B. cereus* (ATCC 14579)	MIC = 700 μg/mL	Florianópolis—SC	[27]
			*S. aureus* (ATCC 6538P)	MIC = 200 μg/mL		
			*S. saprophyticus* (ATCC 35552)	MIC = 200 μg/mL		
			*S. agalactiae* (ATCC 13813)	MIC = 500 μg/mL		
**Coumarins**						
**30**	Tanizin	*Helietta apiculato*	*B. cereus* (ATCC 33019)	MIC = 12.5 μg/mL	Mata—RS	[28]
			*Enterococcus ssp* (ATCC 6589)	MIC = 50 μg/mL		
			*E. aerogenes* (ATCC 13048)	MIC = 25 μg/mL		
			*P. aeruginosa* (ATCC 9027)	MIC = 25 μg/mL		
			*E. coli* (ATCC 25922)	MIC = 50 μg/mL		
			*B. cepacia* (ATCC 17759)	MIC = 25 μg/mL		
			*S. sonnei* (ATCC 25931)	MIC = 25 μg/mL		
			*S. Typhimurium* (ATCC 14028)	MIC = 25 μg/mL		
			*M. morganii* (ATCC 25829)	MIC = 12.55 μg/mL		
**31**	Gravellifenore	*Helietta apiculato*	*B. subtilis* (ATCC 6633)	MIC = 50 μg/ml	Mata—RS	[28]
			*B. cereus* (ATCC 33019)	MIC = 12.5 μg/mL		
			*Enterococcus ssp* (ATCC 6589)	MIC = 50 μg/mL		
			*E. aeruginosa* (ATCC 9027)	MIC = 25 μg/mL		
			*E. coli* (ATCC 25922)	MIC = 50 μg/mL		
			*B. cepacia* (ATCC 17759)	MIC = 3.12 μg/mL		
			*S. sonnei* (ATCC 25931)	MIC = 12.55 μg/mL		
			*S. Typhimurium* (ATCC 14028)	MIC = 50 μg/mL		
			*M. morganii* (ATCC 25829)	MIC = 6.25 μg/mL		
**Phenolic Acids**						
**32**	Gallic acid	*Himatanthus sucuuba*	*S. aureus* (MRSA)	MIC = 31 μg/mL	Santarém—PA	[29]
			*S. epidermidis* (ATCC 12228)	MIC = 31 μg/mL		
			*P. mirabilis* (MRSA)	MIC = 62 μg/mL		
			*S. haemolyticus* (ATCC 2737)	MIC = 62 μg/mL		
			*E. coli* (ATCC 25922)	MIC = 125 μg/mL		
**33**	Protocatechuic acid	*Calophyllum brasiliense*	*B. cereus* (ATCC 14579)	MIC = 500 μg/mL	Florianópolis—SC	[27]
			*S. aureus* (ATCC 6538P)	MIC = 200 μg/mL		
			*S. saprophyticus* (ATCC 35552)	MIC = 200 μg/mL		
			*S. agalactiae* (ATCC 13813)	MIC = 200 μg/mL		
			*E. cloacae* (ATCC 35030)	MIC = 400 μg/mL		
			*E. coli* (ATCC 11775)	MIC = 400 μg/mL		
			*P. aeruginosa* (ATCC 35032)	MIC = 800 μg/mL		
			*P. mirabilis* (ATCC 14273)	MIC = 500 μg/mL		
			*S. Typhimurium* (ATCC 14028)	MIC = 700 μg/mL		
**Other Phenolic Compounds**						
**34**	Dihydrodehydrodiconiferyl alcohol	*Styrax ferrugineus*	*S. aureus* (ATCC 12228)	MIC = 20 μg/mL	Mogi-Guaçu—SP	[30]
**35**	Lyoniresinol	*Clusia burlemarxii*	*S. aureus* (ATCC 6538)	MIC = 25 μg/mL	Mucugê—BA	[19]
**36**	Acid 3,3′-dimethoxyellagic acid-4-*O-*α-rhamnopyranoside	*Euphorbia tirucalli*	*S. aureus* (ATCC 6538)	MIC = 64 μg/mL	Araruna—PB	[16]
			*E. coli* (ATCC 8739)	MIC = 128 μg/mL		
**37**	(E)-methyl-4-hydroxy-3,5-dimethoxycinnamate	*Helietta apiculato*	*B. cereus* (ATCC 33019)	MIC = 12.5 μg/mL	Mata—RS	[28]
			*E. aerogenes* (ATCC 13048)	MIC = 25 μg/mL		
			*B. Ceparia* (ATCC 17759)	MIC = 50 μg/mL		
			*M. morganii* (ATCC 25829)	MIC = 50 μg/mL		
**38**	(E)-ethyl-4-hydroxy-3,5-dimethoxycinnamate	*Helietta apiculato*	*B. cereus* (ATCC 33019)	MIC = 50 μg/mL	Mata—RS	[28]
			*E. aerogenes* (ATCC 13048)	MIC = 50 μg/mL		
**39**	2,2-dimethyl-3,5-dihydroxy-7-(4-hydroxyphenyl) chromane	*Clusia burlemarxii*	*M. luteus* (ATCC 10240)	MIC = 25 μg/mL	Mucugê—BA	[19]
			*S. aureus* (ATCC 6538)	MIC = 50 μg/mL		
			*B. subtilis* (ATCC 6633)	MIC = 100 μg/mL		
			*S. mutans* (ATCC 5175)	MIC = 100 μg/mL		
**40**	Aucuparin	*Kielmeyera coriacea*	*S. aureus* (ATCC 25922)	MIC = 12.5 μg/mL	Mogiguaçu—SP	[25]
			*E. coli* (ATCC 25922)	MIC = 100 μg/mL		
			*B. subtilis* (ATCC 6623)	MIC = 3.12 μg/mL		
			*P. aeruginosa* (ATCC 15442)	MIC = 100 μg/mL		

Caption: * Brazilian States (PB = Paraíba; MG = Minas Gerais; AP = Amapá; BA = Bahia; GO = Goiás; PR = Paraná; SC = Santa Catarina; SP = São Paulo; RS = Rio Grande do Sul; PA = Pará). ** MIC = Minimum inhibitory concentration. *** IC50 = Inhibitory Concentration 50%. **** Substances combined with other antibiotics to measure the synergistic effect are included in parentheses.

## Data Availability

This study consisted of a literature review, and we discussed results previously published in scientific manuscripts. References are included in Table 1.
